# Estimating the time of human decomposition based on skeletal muscle biopsy samples utilizing an untargeted LC–MS/MS-based proteomics approach

**DOI:** 10.1007/s00216-023-04822-4

**Published:** 2023-07-10

**Authors:** Lana Brockbals, Samara Garrett-Rickman, Shanlin Fu, Maiken Ueland, Dennis McNevin, Matthew P. Padula

**Affiliations:** 1 Centre for Forensic Science, School of Mathematical and Physical Sciences, Faculty of Science, University of Technology Sydney, PO Box 123, Broadway, NSW 2007 Australia; 2 School of Life Sciences, Faculty of Science, University of Technology Sydney, PO Box 123, Broadway, NSW 2007 Australia

**Keywords:** Postmortem interval, Human decomposition, Proteomics, Ion mobility separation, Data-independent acquisition, Peptide ratios

## Abstract

Accurate estimation of the postmortem interval (PMI) is crucial in forensic medico-legal investigations to understand case circumstances (e.g. narrowing down list of missing persons or include/exclude suspects). Due to the complex decomposition chemistry, estimation of PMI remains challenging and currently often relies on the subjective visual assessment of gross morphological/taphonomic changes of a body during decomposition or entomological data. The aim of the current study was to investigate the human decomposition process up to 3 months after death and propose novel time-dependent biomarkers (peptide ratios) for the estimation of decomposition time. An untargeted liquid chromatography tandem mass spectrometry–based bottom-up proteomics workflow (ion mobility separated) was utilized to analyse skeletal muscle, collected repeatedly from nine body donors decomposing in an open eucalypt woodland environment in Australia. Additionally, general analytical considerations for large-scale proteomics studies for PMI determination are raised and discussed. Multiple peptide ratios (human origin) were successfully proposed (subgroups < 200 accumulated degree days (ADD), < 655 ADD and < 1535 ADD) as a first step towards generalised, objective biochemical estimation of decomposition time. Furthermore, peptide ratios for donor-specific intrinsic factors (sex and body mass) were found. Search of peptide data against a bacterial database did not yield any results most likely due to the low abundance of bacterial proteins within the collected human biopsy samples. For comprehensive time-dependent modelling, increased donor number would be necessary along with targeted confirmation of proposed peptides. Overall, the presented results provide valuable information that aid in the understanding and estimation of the human decomposition processes.

**Graphical Abstract:**

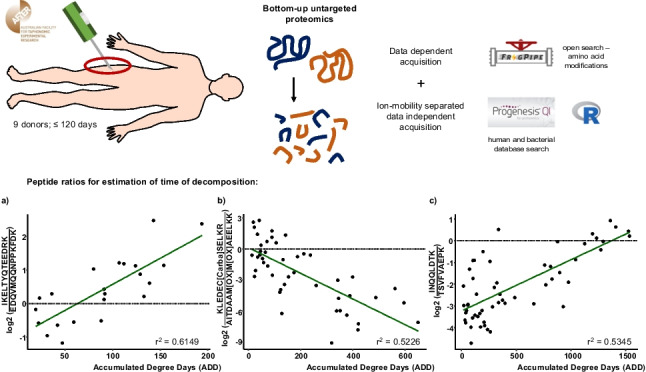

**Supplementary Information:**

The online version contains supplementary material available at 10.1007/s00216-023-04822-4.

## Introduction

Estimation of the time since death is an integral part of forensic medico-legal investigations, particularly in relation to unidentified human remains. Accurate determination of the postmortem interval (PMI) would assist to narrow down an extensive list of missing persons to facilitate positive identification or help to include/exclude an individual from a pool of suspects. Despite its importance, estimation of the PMI remains challenging, due to the complex decomposition chemistry and the variability of extrinsic environmental (e.g. temperature and humidity) and intrinsic factors (e.g. sex, body mass index (BMI) and disease state at time of death) [1–3]. Current methods for PMI estimation often rely on the visual assessment of gross morphological/taphonomic changes of a body during decomposition or entomological data, both of which are known to be highly variable and/or subjective [4, 5]. The use of biochemical techniques in recent years has shown great potential for more objective approaches. Postmortem decomposition is characterised by the chemical breakdown of macromolecules like proteins, lipids and carbohydrates into their structural components [6, 7]. Attempts to identify specific decomposition products for PMI estimation have been made. Examples include (but are not limited to) the analysis of volatile organic compounds (VOCs) [8–11], endogenous metabolites [12–18] and lipids [2, 19]. VOC analyses are crucial to understand the odour profile during soft tissue decomposition to aid in the detection of human remains, but lack a clear correlation with specific PMI [10]. While the potential of endogenous metabolites and lipids to be used for PMI estimation has been shown, studies were based on animal models [13–18] or a small number of human cases [2, 12, 19], which requires further validation before implementation in routine forensic investigations.

Based on the high abundance of muscle tissue within the human body, and its minimally invasive and continuous and easy postmortem accessibility, the use of skeletal muscle for decomposition studies and detection of breakdown products seems advantageous [20]. Indeed, studies investigating protein degradation patterns in skeletal muscle (pig/rat/mouse model and human autopsy cases) in the first few days after death have recently been carried out [20–23]. There is evidence that skeletal muscle protein decay during decomposition is correlated with PMI. However, the studied PMI time-frame is currently limited to the first few days after death and requires additional validation for routine application. For skeletonised human remains, protein biomarkers for PMI and age-at-death estimation have also been proposed, including the assessment of intrinsic and extrinsic variables on the variety and abundance of the bone proteome [24, 25]. While these studies cover the early (fresh and bloated stage) and late (skeletonisation) stages of decomposition, biochemical markers to investigate the active and advanced decay stages are still missing.

Characterisation of the proteome is usually conducted using targeted gel-based (e.g. SDS-PAGE or western blotting) or (un)targeted mass spectrometry–based techniques, with the latter being generally considered as the most sensitive, reliable and high-throughput method [26]. In particular, untargeted liquid chromatography tandem mass spectrometry (LC–MS/MS)–based bottom-up proteomics methods are widely used in exploratory clinical studies for novel biomarker detection, but are currently only sparsely applied to forensically relevant questions [27].

The aim of the current study was to investigate the human decomposition process up to 3 months after death and propose novel time-dependent biomarkers (peptide ratios) for the estimation of decomposition time. An untargeted LC–MS/MS-based bottom-up proteomics workflow was utilized to analyse skeletal muscle collected repeatedly from nine body donors decomposing in an open eucalypt woodland environment in Australia. In addition, general analytical considerations for large-scale proteomics studies for PMI determination are raised and discussed.

## Materials and methods

### Chemical and reagents

Sodium dodecyl sulfate (SDS), Tris–HCl buffer, tris(2-carboxyethyl)phosphine (TCEP), iodoacetamide (IAA), ammonium bicarbonate and LC–MS-grade formic acid were supplied by Sigma-Aldrich (Macquarie Park, NSW, AU). Analytical-grade acetone and ethanol were purchased from ChemSupply (Gillman, SA, AU). Trypsin (gold, mass spectrometry grade) from Promega (Madison, WI, USA) and LC–MS-grade acetonitrile from Honeywell Burdick & Jackson™ (Charlotte, NC, USA) were used. Water was purified to 18.2 MΩ-cm using an Arium® water purification system from Sartorius (Goettingen, DE).

### Sample collection

Sample collection was carried out at the Australian Facility for Taphonomic Experimental Research (AFTER; Sydney, Australia), in an open eucalypt woodland environment [11]. Human donors were obtained through the University of Technology Sydney (UTS) Body Donation Program, giving consent in accordance with the New South Wales Anatomy Act (1997). The study was approved by the UTS Human Research Ethics Committee (ETH15-0029 and ETH18-2999). Nine donors (specific donor information listed in Table 1) were placed on the soil surface between July 2018 and March 2020 and allowed to decompose naturally (median processing time between time of death and placement: 3 days (min.: 1 days; max: 4 days)). This mimics forensically relevant scenarios, and above-ground placement of the bodies allowed for systematic and continuous collection of minimally invasive thigh muscle tissue biopsy samples over the course of 3 months from each donor using a BARD® Magnum™ reusable core biopsy instrument (14-/18-gauge × 10 cm needles; Covington, GA, USA) and puncture wounds were re-sealed with surgical glue after sampling. Where possible, sample collection was carried out every day for the first 7 days after placement, every second day up to 1 month after placement and every fifth day thereafter. Collection was stopped once no soft tissue was retrieved anymore or after 120 days (detailed sample collection time-points per donor are listed in Table S1 within the supplementary material; day 0 refers to day of placement). Individual muscle biopsy samples were lyophilised under negative pressure and stored at − 20 °C until analysis.

**Table 1:** Donor information including sex, body mass, age, season in which decomposition began and the last sample collection time-point in days (with corresponding accumulated degree days (ADD) in brackets; ADD was calculated by addition of average daily temperatures ((minimal daily temperature + maximum daily temperature)/2))

Donor	Sex	Body mass	Age at death	Season in which decomposition began	Last sample collection day (ADD)
Donor 1	Male	Large	85	Winter	110 (1513)
Donor 2	Male	Slim	69	Summer	29 (896)
Donor 3	Male	Slim	87	Summer	13 (337)
Donor 4	Female	Medium	88	Autumn	120 (1533)
Donor 5	Female	Medium	63	Autumn	118 (1492)
Donor 6	Male	Slim	74	Winter	117 (1525)
Donor 7	Female	Slim	82	Spring	24 (539)
Donor 8	Female	Slim	97	Spring	10 (240)
Donor 9	Female	Large	75	Autumn	75 (1167)

### Sample preparation

Lyophilised samples were manually homogenised on dry ice and powdered tissue re-suspended in 500 µL of 1% SDS in 100 mM Tris–HCl (pH 8.8) for protein extraction. Samples were sonicated (10 s at 70% intensity), boiled (10 min at 95 °C) and centrifuged (5 min at 20,000 g). TCEP and IAA were added to a final concentration of 5 mM and 10 mM, respectively, to the supernatant and incubated at room temperature for 1 h. Proteins were precipitated by addition of 2.5 mL ice-cold acetone and stored at − 20 °C overnight. After centrifugation (5 min at 20,000 × g), the supernatant was discarded and the resulting pellet re-suspended in 250 µL of 1% SDS in 100 mM Tris–HCl (pH 8.8). Samples were normalised to 40 µg of protein (quantification using Pierce™ BCA Protein Assay Kit) and purified using single-pot solid-phase-enhanced sample preparation (SP3) adapted from Hughes et al. [28]. In short, a paramagnetic bead suspension was added to all samples (SpeedBeads™ magnetic carboxylate modified particles, Merck, Germany; bead concentration 0.5 µg/µL) along with an equal volume of 100% ethanol, and incubated at 24 °C for 5 min with shaking. In a magnetic rack, beads were aggregated on the tube wall and the supernatant was discarded. Magnetic beads were washed with 180 µL of 80% ethanol three times (5 min incubation and supernatant discarded each time) before re-suspension in 100 µL ammonium bicarbonate (100 mM) and subsequent digestion with trypsin (1:40 ratio) at 37 °C overnight. Samples were then centrifuged and transferred into glass inserts for LC–MS/MS analysis.

### Liquid chromatography–high resolution mass spectrometry

For full scan analysis with data-independent acquisition (DIA), an Acquity M-class nanoLC system (Waters, Milford, MA, USA) was used, coupled to a Synapt XS time-of-flight (TOF) MS (Waters, Milford, MA, USA). One microliter of sample material was loaded onto a nanoEase Symmetry C18 trapping column (180 µm × 20 mm) with eluent A (0.1% formic acid in water; 10 µL/min, 1 min), before being washed onto an HSS T3 column (75 µm × 150 mm) heated at 50 °C with a flow rate of 0.5 µL/min. Gradient elution was achieved with eluent A and eluent B (ACN), starting at 1% B, increased to 40% B within 46 min, further increased to 85% B after 48 min, held for 2 min and equilibrated at the starting conditions (1% B) for the remainder of the acquisition time. The total run time per sample was 55 min. The MS was operated with an electrospray ion source in positive mode with an ionisation voltage of 3 kV at a fixed cone voltage of 20 V. Within the performed ultra-definition MS^E^ (UDMS^E^) experiment, peptide ions were first separated by travelling wave ion mobility spectrometry (TWIMS) at a transfer wave velocity of 155 m/s, applying a charge state/drift time stripping rule file to remove 1 + ions prior to collision induced dissociation (CID) energy scan. After mobility separation, peptides were subjected to alternating low-energy (6 eV; no fragmentation) and high-energy CID with an accumulation time of 0.4 s for each scan type. High-energy CID was performed in the transfer cell using a look-up table adapted from Distler et al. [29]. TOF scans were performed over the mass range of 50–1500 m*/z*. All samples were analysed in triplicates in a randomised order to increase robustness.

In addition, a single injection of all samples was run in data-dependent acquisition (DDA) mode on an Acquity M-class nanoLC system (Waters, Milford, MA, USA) coupled to a Thermo Scientific Q Exactive Plus orbitrap MS (Bremen, Germany). Five microliters of the sample was first loaded onto a nanoEase Symmetry C18 trapping column (180 mm × 20 mm; at 15 µL/min for 3 min) before being washed onto a nanobore column with an integrated emitter manufactured with a laser puller (75 mmID × 350 mm) packed in-house with SP-120–1.7-ODS-BIO resin (1.7 mm, Osaka Soda Co, Osaka, JP). The column was heated to 45 °C during peptide separation. The following elution gradient was run using eluent A (0.1% formic acid in water) and B (ACN): 5–30% eluent B over 90 min, increased to 30–80% eluent B over 3 min, held for 2 min and equilibrated at the starting conditions (5% B) for the remainder of the acquisition time. The total run time per sample was 150 min. Eluted peptides were ionised in positive electrospray ionisation mode at 2.4 kV. A survey scan was performed between 350 and 1500 Da at 70,000 resolution for peptides of charge state 2 + or higher with an AGC target of 3e^6^ (max injection time: 50 ms). The top-12 peptides were selected for fragmentation in the HCD cell using an isolation window of 1.4 m*/z*, an AGC target of 1e^5^ and maximum injection time of 100 ms. Fragments were scanned in the orbitrap analyser at 17,500 resolution and the product ion fragment masses measured over a mass range of 120–2000 Da. The mass of the precursor peptide was then excluded for 30 s. All samples were analysed in randomised order.

### Data processing

DDA data was processed using the open search algorithm of FragPipe (default processing parameters) [30, 31]. The aim was to identify amino acid modifications within the dataset to be included in the subsequent ion-mobility DIA data search to improve proteome coverage.

Progenesis Qi for proteomics (Nonlinear Dynamics, Milford, MA, USA) was used for ion-mobility DIA data processing and peptide/protein identification. The current dataset consisted of triplicate analyses of 171 sampling time-points. In summary, data was lockmass corrected (*m/z* 785.8426), peak picked (max charge: 7), retention time (RT)–aligned (RT limits: 10–53 min) and searched against the reviewed human database allowing for 2 missed cleavages and the following variable modifications: carbamidomethyl (C), deamidation (N), deamidation (Q), oxidation (M) and oxidation (P). Relative protein quantification was achieved using top-3 peptides. Normalised peptide abundances (normalised to all proteins) and identifications were exported for further processing using R (version 4.2.1, package: tidyverse) [32, 33]. Mean peptide abundances over technical replicates were calculated and filtered out if < 1000 as these were suspected background noise. Additionally, peptides/proteins without positive identification were excluded. All possible peak area ratios (normalised) between peptide features originating from the same protein (ordered by peptide sequence length) were calculated, and linear regression of each individual log2 transformed ratio against decomposition time was performed as adapted from Schneider et al. [34]. For this purpose, all sample collection time-points [days] were converted to accumulated degree days (ADD) by addition of average daily temperatures ((minimal daily temperature + maximum daily temperature)/2) [35]. This allowed comparability between donors placed in different seasons/temperatures across a 2-year period (July 2018 to June 2020) and decreased variability. Database matches of peptide ratios were manually confirmed within Progenesis Qi for proteomics if during linear regression, ratios showed a coefficient of determination (*r*^2^) of ≥ 0.5, a slope ≥ 0.001 or ≤  − 0.001 and if within each subgroup, a ratio was able to be calculated for more than 40% of the collection time-points (i.e. not excluded by previous filter criteria). Subgroups studied were decomposition < 200 ADD (*n* = 61 collection time-points), decomposition < 655 ADD (*n* = 122 collection time-points), decomposition < 1535 ADD (*n* = 171 collection time-points), donors with a body mass classified as slim (*n* = 5 donors), donors with a body mass classified as medium/large (*n* = 4 donors), female donors (*n* = 5 donors), and male donors (*n* = 4 donors). Time-dependent subgroups were chosen to represent short (≤ 15 days of decomposition), medium (≤ 52 days) to long-term (≤ 120 days) decomposition ranges. Body mass at time of death was approximated by mortuary staff prior to arrival at AFTER.

In a second phase, the ion-mobility DIA dataset was re-processed using the same parameters as detailed above, but searched for bacterial peptide identifications. For this, a database was created that included bacteria strains involved in postmortem processes (postmortem gut microbiome and adipocere formation) according to previous literature [36, 37]. In total, reviewed protein identifications for 26 bacterial strains were included (complete list of bacterial strains can be found within the supplementary material Table S2).

## Results and discussion

### Analytical considerations

One of the initial key decisions that must be made when designing a proteomics experiment is the choice of acquisition method. While DDA used to be most commonly utilized for bottom-up proteomics, limitations, such as stochastic precursor ion selection and length of cycle times, led to the development of DIA strategies that result in complex but comprehensive product ion data [29, 38]. Classically, DIA data is searched against a spectral library, created from DDA data, for peptide/protein identification. Advances in software algorithms, however, also allow library-free searches using conventional databases of the studied organism for identification (e.g. DIA-NN or FragPipe) [39, 40]. In combination with ion mobility separation, DIA offers reproducibility of data and extensive proteome coverage, making it more suitable as the main mode of analysis within the current study. One of the main challenges that was encountered, however, was the availability of software solutions that would allow processing of ion-mobility separated DIA data in a Waters.raw file format using library-free searches. For the current study, a commercially available software (Progenesis Qi for proteomics, Nonlinear Dynamics, Milford, MA, USA) had to be used for initial data processing (e.g. peak picking, RT alignment and peptide/protein identification using a database search) instead of an open-source workflow. Based on the complex nature of postmortem data and the unpredictability of postmortem processes, an open search for amino acid modifications would have been more ideal to identify unconventional time-dependent postmortem modifications. It was not possible, however, to use the open search algorithm of FragPipe with the acquired ion-mobility DIA dataset (neither in Waters.raw file format nor converted to an open file format) [30, 31]. Hence, it was decided to additionally analyse all samples with a DDA method to be able to perform this processing step. Based on the extended run time (150 min), it was only feasible to run a single injection of all samples in DDA mode. Ion-mobility DIA data from other vendors e.g. Thermo Scientific or Bruker seem to be more widely supported in this context. In general, the open search processing step using the DDA dataset did not yield any unexpected findings. As detailed in Table 2, 80% of peptide-spectrum matches did not show any modifications. Mass shifts for deamidation, oxidation/hydroxylation and dihydroxylation occurred in at least 1% of the peptide spectra. Combined with their presumed localisation (in addition to their commonly observed localisation), it was decided to include deamidation (asparagine and glutamine) and oxidation (proline and methionine) along with sample preparation-induced carbamidomethyl (cysteine) as variable modifications for the database search of ion-mobility DIA data. A dihydroxylation mass shift for peptides did not show a strong localisation to a specific amino acid and was therefore most likely the result of two individual oxidation events.

**Table 2:** Results of the open search for modifications occurring within the peptide-spectrum matches of the DDA dataset

Modification	% of peptide-spectrum matches within the dataset	Δ mass (monoisotopic)	Amino acid localisation
None	80	-	-
Deamidation	4.7	0.9840	N (asparagine)
Oxidation or hydroxylation	1.2	15.9949	P (proline)
Dihydroxylation	1.0	31.9898	No strong localisation

Another aspect that should be considered, particularly when conducting a large-scale untargeted proteomics study with complex (ion-mobility) DIA data, is the availability of a powerful processing computer. The total dataset consisted of triplicate analyses of 171 sampling time-points, resulting in more than 500 samples to be processed within the same batch to achieve RT alignment. Progenesis Qi for proteomics required 256 GB random-access memory (RAM) to be able to process the dataset. Additionally, it was found that the use of a powerful graphics card could help improve the speed of specific processing tasks. Data post-processing and statistical analyses were performed outside the core processing software using R or Python to allow full customisation.

### Generalised time-dependent peptide ratios

Within the complete dataset, comprised of 171 sample collection time-points across 9 donors, a total of 161,026 features were detected, of which 21,153 could be positively identified as peptides that had a normalised abundance greater than 1000. Utilizing the post-processing workflow detailed above, 1,874,191 ratios between peptides that originate from the same protein were calculated. This concept was adapted from Schneider et al. [34] and can be classified as a pseudo internal standard normalisation strategy. Of these calculated peptide ratios only a very small number of ratios also satisfied the filter criteria after linear regression (*r*^2^ ≥ 0.5, slope ≥ 0.001 or ≤  − 0.001, occurrence in more than 40% of the collection time-points per subgroup, database match positively confirmed manually). As listed in Table 3, six peptide ratios show promising linear regression correlation for the time-frame smaller than 200 ADD; a further 4 peptide ratios for the time-frame smaller than 655 ADD. No peptide ratio satisfied the applied filter criteria for the time-frame smaller than 1535 ADD. As the subgroup < 1535 ADD included the complete dataset with all 171-sample collection time-points across all 9 donors, it was decided to also include peptide ratios that only occur in more than 30% of all possible time-points. Following this, two promising peptide ratios were identified that had more than 51 datapoints across all nine studied donors. While these two peptide ratios (INQQLDTK/LYDQHLGK and INQQLDTK/TSVFVAEPK) seemed promising for a generalised application in PMI estimation, the underlying peptides were found to have multiple possible protein origins, hence are not unique for a single protein. This could have an influence on the repeatability of results, as the intended pseudo internal standard normalisation that was the basis for the ratio calculation (described above) could be compromised by this. However, looking at the suggested protein origin, the presumed origin is myosin-2 with myosin-1, myosin-3, myosin-4, myosin-8 and myosin-13, listed as alternative origins. Indeed, myosin-2 seems to be the most likely origin for the listed peptides, as after myosin-7, myosin-2 is the most abundant myosin isoform present within human skeletal muscle tissue (regional and muscle-specific differences exist) [41, 42]. Based on this, both peptide ratios INQQLDTK/LYDQHLGK and INQQLDTK/TSVFVAEPK were retained as promising indicators for time of death estimation (exemplified mass spectra for these three peptides can be found in Figure S1 of the supplementary material). The same holds true for the peptide ratios TIHELEK/LTGAIMHFGNMK (subgroup < 200 ADD) and KLEDEC[Carba]SELKR/AITDAAM[OX]M[OX]AEELKK (subgroup < 655 ADD), with myosin-7 being the most likely protein origin, compared to alternative myosin isoforms.

**Table 3:** Peptide ratios suggested for estimation of three different postmortem periods (< 200 accumulated degree days (ADD), < 655 ADD and < 1535 ADD; listed are the mass-to-charge ratios (*m/z*) and retention times (RT) of each peptide along with the amino acid sequence, protein origin (with alternative possible origin listed in brackets) and the results of the linear regression (coefficient of determination (*r*^2^) and slope); [Carba]: carbamidomethyl modification; [OX]: oxidation modification

	Feature ratio [m/z_RT]	Peptide ratio	Protein origin	*r*^2^	Slope
200 ADD	413.9671_19.8/979.5045_30.8	IKELTYQTEEDRK/ KALQEAHQQALDDLQAEEDKVNTLTK	Myosin-7	0.5739	0.0154
	667.8613_33.9/946.1713_44.7	M[OX]QLLEIITTEK/KLDSLTTSFGFPVGAATLVDEVGVDVAK	Trifunctional enzyme subunit alpha, mitochondrial	0.5425	− 0.0087
	762.8820_21.7/568.6068_22.7	IKELTYQTEEDR/ EDQVMQQNPPKFDK	Myosin-7	0.5095	0.0157
	762.8820_21.7/979.5045_30.8	IKELTYQTEEDR/ KALQEAHQQALDDLQAEEDKVNTLTK	Myosin-7	0.5023	0.0152
	826.9289_20.0/568.6068_22.7	IKELTYQTEEDRK/ EDQVMQQNPPKFDK	Myosin-7	0.6149	0.0154
	869.4724_17.1/660.3376_30.4	TIHELEK/ LTGAIMHFGNMK	Myosin-7 (myosin-3, myosin-15)	0.5557	0.0128
655 ADD	1153.0520_30.3/774.3700_28.0	LTQESIMDLENDKQQLDER/LTQESIM[OX]DLENDKQQLDER	Myosin-7	0.5139	− 0.0079
	469.5694_18.3/518.5909_23.8	KLEDEC[Carba]SELKR/ AITDAAM[OX]M[OX]AEELKK	Myosin-7 (myosin-2, myosin-1, myosin-6, myosin-4, myosin-8, myosin-3, myosin-13, myosin-7b)	0.5226	− 0.0124
	636.7858_19.4/774.3700_28.0	TLEDQMNEHR/ LTQESIM[OX]DLENDKQQLDER	Myosin-7	0.5007	− 0.0087
	709.8910_24.7/736.8491_40.1	KKDFELNALNAR/QLEAEKM[OX]ELQSALEEAEASLEHEEGK	Myosin-7	0.5035	− 0.0072
1535 ADD	959.5157_17.4/487.2571_18.5	INQQLDTK/ LYDQHLGK	Myosin-2 (myosin-1, myosin-4, myosin-8, myosin-3, myosin-13)	0.5266	0.0016
	959.5157_17.4/489.2682_24.3	INQQLDTK/ TSVFVAEPK	Myosin-2 (myosin-1, myosin-4, myosin-8, myosin-3, myosin-13)	0.5345	0.0024

Overall, 12 time-dependent peptide ratios for estimation of three different postmortem periods were identified as listed in Table 3. All but one peptide ratio pair originated from myosin isoforms. In striated muscle, myosin filaments overlap with thin, actin-containing filaments that make up the sarcomeres (basic unit for contraction) [43]. As the basis for this study was the analysis of thigh muscle tissue samples, this result is not surprising and in fact underlines the suitability of the study design. Additionally, one promising peptide ratio (M[OX]QLLEIITTEK/KLDSLTTSFGFPVGAATLVDEVGVDVAK, subgroup < 200 ADD) was calculated using two peptides from the protein P40939 (Trifunctional enzyme subunit alpha, mitochondrial). Again, its occurrence in muscle tissue is expected, as the mitochondrial trifunctional enzyme catalyses the last three of the four enzymatic reactions during the beta-oxidation pathway, the major energy production process in tissues during which fatty acids are broken down to acetyl-CoA [44].

**Fig. 1:**
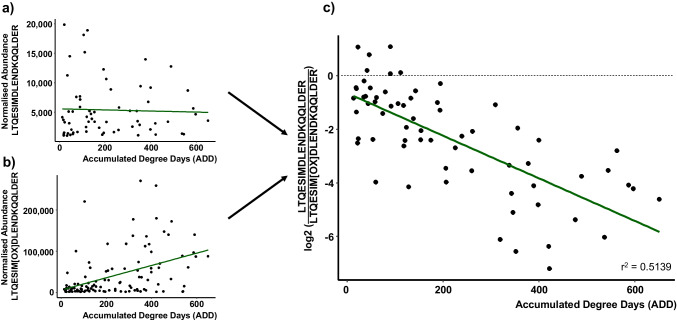
Normalised abundances of peptides LTQESIMDLENDKQQLDER (**a**) and LTQESIM[OX]DLENDKQQLDER (**b**) over time as well as log2 transformed peptide ratio LTQESIMDLENDKQQLDER/LTQESIM[OX]DLENDKQQLDER (**c**) over 655 accumulated degree days (ADD); displayed trendlines represent linear regression results; [OX]: oxidation modification

An interesting promising peptide ratio within the < 655 ADD subgroup is LTQESIMDLENDKQQLDER/LTQESIM[OX]DLENDKQQLDER. Here, a ratio between the non-oxidised peptide and the peptide with an oxidised methionine was calculated. As displayed in Fig. 1, the normalised abundance of LTQESIMDLENDKQQLDER does not seem to significantly change over time. In contrast, the oxidised form (LTQESIM[OX]DLENDKQQLDER) shows higher normalised abundances over time and the increased occurrence of oxidation reactions could be used as a marker for ongoing decomposition processes. Previous studies also found amino acid modifications, including the oxidation of methionine, as potential markers for the determination of the time since deposition of a blood spot [34]. Additionally, deamidation processes of asparagine and glutamine residues in archaeological samples have been proposed as an indicator of thermal age and for relative dating [45].

**Fig. 2:**
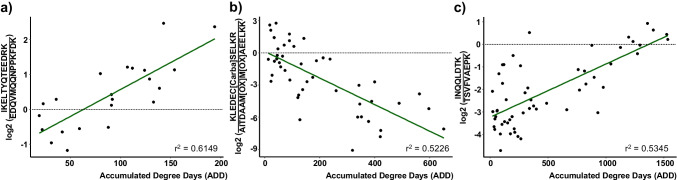
Peptide ratios with the strongest coefficient of determination (*r*^2^) per subgroup; **a** peptide ratio IKELTYQTEEDRK/EDQVMQQNPPKFDK for < 200 accumulated degree days (ADD), **b** peptide ratio KLEDEC[Carba]SELKR/AITDAAM[OX]M[OX]AEELKK for samples < 655 ADD, **c** peptide ratio INQQLDTK/TSVFVAEPK for the complete dataset (< 1535 ADD); displayed trendlines represent linear regression results; [Carba]: carbamidomethyl modification; [OX]: oxidation modification

The ratios with the strongest coefficient of determination (*r*^2^) per subgroup, a statistical measure of how well the regression line approximates the actual data, are shown in Fig. 2. For sample collection time-points smaller than 200 ADD, the peptide ratio IKELTYQTEEDRK/EDQVMQQNPPKFDK seems to be best suited to distinguish between early PMIs with *r*^2^ = 0.6149. Within the current dataset, 200 ADD was reached at least after day 15 of sample collection and as early as after day 5 (depending on the season of decomposition), making this ratio a potential biomarker for the first few days of decomposition. The peptide ratio KLEDEC[Carba]SELKR/AITDAAM[OX]M[OX]AEELKK showed a negative slope with an *r*^2^ of 0.5226 for subgroup < 655 ADD, while the peptide ratio INQQLDTK/TSVFVAEPK (*r*^2^ = 0.5345, subgroup < 1535 ADD) exhibited a positive slope. Both peptide ratios exhibited promising exponential postmortem time-dependent behaviour that could be used for estimating decomposition time in the medium (< 52 days of decomposition) to long-term range (< 120 days of decomposition). To extend the postmortem time-frame beyond 120 days, a longer study would need to be designed. However, the basis for the current method is the collectability of muscle tissue samples, which is often not available past 3 months of decomposition, particularly with warmer environmental temperatures. In general, to distinguish between the use of the proposed overall peptide ratios (subgroup < 1535 ADD) and the proposed subgroups ratios (< 200 ADD/ < 655 ADD) within a forensic investigation, previous temperature data for the region where the body was found would be required. This can be achieved retrospectively, but in addition, a rough estimation of the decomposition time would be needed to place a deceased with an unknown time of death into one of the three proposed postmortem time-frames (< 200 ADD, < 655 ADD or < 1535 ADD). This could be accomplished by evaluation of the morphological changes of decomposition. This, however, is highly subjective and error prone, particularly due to differential decomposition [11, 46]. Therefore, if muscle tissue samples can be collected from a deceased, it may be prudent to use the two peptide ratios derived from myosin-2 (subgroup < 1535 ADD). Knowledge of case circumstances could still warrant the use of the shorter postmortem time-frame ratios.

### Time-dependent peptide ratios separated by intrinsic factors (sex, body mass)

For applicability in routine forensic investigations, the aim should be to find generalised peptide ratio markers that can be used equally in all case circumstances. The number of donors in the current study, however, is limited and might not be statistically large enough to account for all intrinsic cofounding factors (e.g. sex, body mass and disease state at time of death). Indeed, while temperature is a major extrinsic environmental factor that significantly impacts decomposition rates, it is also suggested that intrinsic factors may impact microbial activity and lead to inter-individual variability in decomposition patterns [47, 48]. Hence, the current dataset was divided according to the intrinsic factors sex (female and male) and body mass (slim and medium/large) and the subsets discussed individually. For simplicity, only peptide ratios with a unique protein of origin were considered.

#### Female vs. male

The current dataset included five female donors, three of which started decomposition in autumn (sampled for 75, 118 and 120 days respectively). The other two donors exhibited rapid decomposition starting in spring, leading to sample collection being stopped after 10 and 24 days, respectively. Peptide ratios were calculated and linear regression analysis performed across the entire postmortem period studied (i.e. < 1535 ADD). Listed in Table 4 are the two promising peptide ratios found within the female subgroup. Both peptide ratios originate from myoglobin, a protein that facilitates movement of oxygen within the muscle and serves as a reserve supply of oxygen [49]. Additionally, four male donors were included in the current study. Two started to decompose in the Australian winter months (sample collection carried out until days 110 and 117, respectively), whereas the other two donors were placed for decomposition in summer (last sample collection days were days 13 and 29, respectively). Identical to the female subgroup, ratio calculation and regression analysis was carried out over the complete postmortem time-frame of the study (< 1535 ADD). Thirteen peptide ratios were identified that satisfied all specified filter criteria (see Table 4). Similar to the generalised time-dependent peptide ratios, 11 of these have a myosin isoform as their unique protein origin (myosin-1 and myosin-2) and included oxidative amino acid modifications (e.g. peptide KLETDISQMQGEMEDILQEAR with two different oxidation modifications methionine 1 [OX] and methionine 2 [OX]). This supports the points raised within the previous section that the increased occurrence of oxidation reactions could potentially be used as a marker for ongoing decomposition processes. Additionally, the peptide ratio QKYDITTLR/ELWETLHQLEIDKFEFGEK (*r*^2^ = 0.5644) originates from the protein troponin T (fast skeletal muscle; P45378). This is a striated muscle-specific protein and serves as the tropomyosin-binding subunit of troponin [50]. Previous studies showed that cardiac troponin T started to degrade after a few days after death in pig muscle tissue [20, 22]. Following this, observing postmortem time-dependent changes on the peptide level in the current study also supports the potential of troponin T as an indicator for decomposition time. Furthermore, one peptide ratio with a promising exponential postmortem behaviour (*r*^2^ = 0.6221) originated from glycogen phosphorylase (muscle form; P11217; TIFKDFYELEPHK/VHINPNSLFDIQVK). This enzyme plays a crucial role in facilitating rapid energy delivery for contraction in the muscle, by breaking down the glycogen polymer bonds to release glucose molecules [51]. Overall, it is not surprising that sex-specific peptide ratios were identified, as sexual dimorphism of skeletal muscle is well known. Differences in gene expression, presumably mediated by hormone levels, for example, lead to a larger muscle mass in men compared to women [52]. Hence, for applicability of these peptide ratios during forensic investigations, the sex of the deceased would need to be determined. While in the early decomposition stages this might seem achievable but ongoing decomposition and medical gender-affirming surgery may complicate this process and may lead to wrong classifications. Use of generalised time-dependent, sex-independent peptide ratios are therefore advised, unless knowledge of case circumstances warrant the use of sex-specific peptide ratios.

**Table 4:** Peptide ratios suggested for estimation of decomposition time based on the intrinsic factors sex (female and male) and body mass (slim and medium/large); listed are the mass-to-charge ratios (*m/z*) and retention times (RT) of each peptide along with the amino acid sequence, protein or origin and the results of the linear regression (coefficient of determination (*r*^2^) and slope); [OX]: oxidation modification

	Feature ratio [m/z_RT]	Peptide ratio	Protein origin	*r*^2^	Slope
Female	374.7219_30.6/900.9550_38.6	ALELFR/ GLSDGEWQLVLNVWGK	Myoglobin	0.5564	− 0.0032
	650.3148_25.8/900.9550_38.6	ELGFQG/ GLSDGEWQLVLNVWGK	Myoglobin	0.5206	− 0.0031
Male	379.8810_23.5/797.7348_39.9	QKYDITTLR/ ELWETLHQLEIDKFEFGEK	Troponin T, fast skeletal muscle	0.5644	0.0032
	394.2364_18.9/585.2761_17.6	NTQAILK/ LQTESGEYSR	Myosin-1	0.5465	0.0035
	509.7801_26.9/749.0445_30.0	DTLVSQLSR/ TEAGATVTVKDDQVFPMNPPK	Myosin-1	0.5223	− 0.0028
	556.2885_32.5/812.4450_35.1	TIFKDFYELEPHK/ VHINPNSLFDIQVK	Glycogen phosphorylase muscle form	0.6221	− 0.0013
	577.2793_20.4/440.5622_21.7	LQTESGEFSR/ LTGAVM[OX]HYGNLK	Myosin-2	0.5260	− 0.0023
	577.2793_20.4/827.3936_32.7	LQTESGEFSR/KLETDISQMQGEM[OX]EDILQEAR	Myosin-2	0.5233	− 0.0038
	585.2761_17.6/759.8777_24.7	LQTESGEYSR/ IEDEQALGM[OX]QLQK	Myosin-1	0.5042	− 0.0033
	585.2761_17.6/821.4081_33.5	LQTESGEYSR/KLETDISQIQGEM[OX]EDIIQEAR	Myosin-1	0.6312	− 0.0050
	644.8354_19.5/827.3942_34.5	IEAQNRPFDAK/KLETDISQM[OX]QGEMEDILQEAR	Myosin-2	0.6111	− 0.0035
	787.4676_18.9/573.3061_27.8	NTQAILK/ ALEDQLSEIK	Myosin-1	0.5184	0.0020
	787.4676_18.9/585.2761_17.6	NTQAILK/ LQTESGEYSR	Myosin-1	0.5277	0.0036
	825.8991_32.2/827.3936_32.7	MEIDDLASNVETVSK/KLETDISQMQGEM[OX]EDILQEAR	Myosin-2	0.5593	− 0.0036
	849.8828_29.7/821.4081_33.5	MEIDDLASNMETVSK/KLETDISQIQGEM[OX]EDIIQEAR	Myosin-1	0.5364	− 0.0027
Slim	356.6818_16.1/859.4289_45.4	YSESVK/ AISEELDNALNDITSL	Tropomyosin beta chain	0.5756	0.0038
	714.3460_30.6/590.8140_27.6	TLYGFGG/ ISGLIYEETR	Histone H4	0.5234	− 0.0080

#### Body mass slim vs. medium/large

Within the current dataset, four donors were classified as medium/large (all decomposing in winter/autumn), the remaining five donors were classified as slim. Of the slim donors, only one started decomposition in winter (last sample collection after 117 days), whereas four donors were placed in summer/spring (sample collection possible until days 10, 13, 24 and 29 respectively). Following this, the dataset of the subgroup “body mass slim” could be skewed towards very rapid decomposition. However, it was still investigated over the complete studied postmortem time-frame (< 1535 ADD) for greatest possible comparability. Indeed, two promising body mass-specific peptide ratios could be identified for slim donors (see Table 4). These originated from the tropomyosin beta chain (P07951; which modulates actin-myosin interaction and regulates contractility in striated muscle [53]) and histone (H4; P62805; core component of nucleosomes). In contrast, no peptide ratio could be found for the subgroup “body mass medium/large” that satisfied all defined filter criteria (see material and methods section). The BMI was previously highlighted as a potential intrinsic factor for inter-individual differences in the postmortem microbiome and decomposition patterns [47, 48]. It was therefore surprising to not be able to identify more body mass-specific peptide ratios within the current dataset. Regardless, routine applicability of body mass-specific peptide ratios for estimation of time of decomposition would be challenging. With ongoing decomposition, the body mass at time of death would be difficult to estimate retrospectively and would be prone to errors.

### Bacterial search

The analysed data were re-processed in a second step to identify bacterial peptides present in the muscle tissue samples. The hypothesis was that with ongoing decomposition, bacterial processes become dominant and their time-dependent changes could also be used to estimate decomposition time. From the total number of features (161,026), only 1061 were successfully identified as bacterial peptides originating from the defined strains. From these, 10,252 peptide ratios (a combination of 355 different peptides) were calculated and linear regression analysis performed. Unfortunately, the identity of only 10 peptides could be successfully confirmed during manual checking of the database matches. Following this, only one out of 10,252 peptide ratios remained that satisfied all filter criteria: AQIEEIASDIER/AQIEEIASDIER; m/z_RT 1373.6896_27.3/687.3497_27.3. Upon closer inspection, it was found that this ratio was calculated from the same peptide in two different charge states (1 + /2 +), and was only present in 6 samples out of 171, so this was discarded as a possible peptide ratio. Overall, the search against a bacterial database did not yield any supportive results for the estimation of decomposition time. A potential explanation was recently highlighted by Aziz et al. [54], who searched for viral proteins in human gastric biopsy samples. Similarly to the current study, they found a low number of viral proteins successfully being assigned, almost half of which also had sequence homology to human proteins and should therefore being used with caution. They reasoned that viral proteins as a minor species do not dominate the human biopsy sample proteome which analytically leads to poor spectral quality for the derived peptides. This in turn leads to weak database matches, as the matching algorithm has difficulties with low signal-to-noise ratios. Although the current study used DIA data, which should lead to a broad coverage of the proteome, this is a very likely explanation and underlines the difficulty for future studies to use LC–MS/MS-based proteomics approaches to detect bacterial decomposition marker in human tissue samples.

## Limitations and conclusions

The current study proposes peptide ratios as a first step towards biochemical estimation of the decomposition time within the first 120 days after death (< 1535 ADD, < 655 ADD, < 200 ADD, female donors, male donors and slim donors). This is crucial to aid in an objective estimation of the time of death in forensic investigations, particularly in the context of finding unknown human remains. One of the main limitations of the current study is the fact that the time and storage/transport conditions between death and first sampling time-point after placement of the donor could not be controlled and varies between donors (max. 4 days). This could not be circumvented as this study relied on voluntary donations to the UTS Body Donation Program. Therefore, sample collection time-point 0 within this study always refers to the time of placement of the donor and not to time of death. Hence, time-interval of decomposition is discussed rather than actual postmortem interval. Before the proposed peptide ratios can be applied to forensic case work, it is necessary to significantly increase the number of donors to confirm robustness of the peptide ratios across a multitude of extrinsic and intrinsic conditions (including burials). This would also help to draw more generalised conclusions and conduct a comprehensive modelling approach. Additionally, targeted method development for the proposed promising peptides is required to confirm the results of this untargeted, shotgun proteomics workflow, including the confirmation of the proposed peptides using synthetically produced reference materials (e.g. matching fragmentation patterns and RT). Once this is achieved, targeted peptide analysis can be validated according to international guidelines. Available RAM is a limiting factor for proteomic analysis and 64 GB RAM were not sufficient for the dataset in the current study. When conducting large-scale untargeted proteomics with complex (ion mobility separated) DIA data, powerful computation is required and data processing strategies should be thoroughly considered before data acquisition to ensure the availability of suitable processing software and computer systems. Overall, the results of this study provide valuable information that can aid in the understanding and estimation of the human decomposition processes.

## Supplementary Information

Below is the link to the electronic supplementary material.

Supplementary file1 (DOCX 4760 KB)

## Declarations

### Source of biological material

Minimally invasive muscle biopsy sample collection was carried out at the Australian Facility for Taphonomic Experimental Research (AFTER; Sydney, Australia). Human donors were obtained through the University of Technology Sydney (UTS) Body Donation Program, giving consent in accordance with the New South Wales Anatomy Act (1997).
