# Supplementary material for: Estimating the time of human decomposition based on skeletal muscle biopsy samples utilizing an untargeted LC–MS/MS-based proteomics approach
Source: Anal Bioanal Chem. 2023 Jul 10;415(22):5487–98. doi: 10.1007/s00216-023-04822-4 (PMC10444689; doi:10.1007/s00216-023-04822-4)
Supplement: Supplementary file 1 — Supplementary file1 (DOCX 4760 KB) [file 216_2023_4822_MOESM1_ESM.docx]

**Supplementary Material**

**Estimating the time of human decomposition based on skeletal muscle biopsy samples utilizing an untargeted LC-MS/MS/based proteomics approach**

Lana Brockbals ^1+^, Samara Garrett-Rickman ^1+^, Shanlin Fu ^1^, Maiken Ueland ^1^, Dennis McNevin ^1^, Matthew P Padula ^2^*

1) Centre for Forensic Science, School of Mathematical and Physical Sciences, Faculty of Science, University of Technology Sydney, PO Box 123, Broadway 2007 NSW, Australia

2) School of Life Sciences, Faculty of Science, University of Technology Sydney, PO Box 123, Broadway 2007 NSW, Australia

+ joint co-first authors

* corresponding author; E-Mail address: Matthew.Padula@uts.edu.au

OrcIDs:

Lana Brockbals: 0000-0001-7310-2671

Samara Garrett-Rickman: 0000-0002-8000-8488

Shanlin Fu: 0000-0002-6238-3612

Maiken Ueland: 0000-0002-9155-3502

Dennis McNevin: 0000-0003-1665-3367

Matthew P Padula: 0000-0002-8283-0643

Table S1: Detailed list of sampling time-points [day] per donor (after placement) and associated accumulated degree days (ADD); Day 0 refers to day of placement; ADD was calculated by addition of average daily temperatures ((minimal daily temperature + maximum daily temperature) / 2).

| Donor number | Sampling time-point [day] | ADD |
| --- | --- | --- |
| 1 | 1 | 24 |
|  | 30 | 343 |
|  | 31 | 356 |
|  | 33 | 378 |
|  | 34 | 390 |
|  | 35 | 400 |
|  | 38 | 438 |
|  | 42 | 489 |
|  | 48 | 563 |
|  | 50 | 587 |
|  | 56 | 656 |
|  | 60 | 704 |
|  | 65 | 773 |
|  | 70 | 855 |
|  | 75 | 938 |
|  | 85 | 1086 |
|  | 91 | 1186 |
|  | 100 | 1325 |
|  | 105 | 1413 |
|  | 110 | 1513 |
| 2 | 0 | 31 |
|  | 3 | 119 |
|  | 8 | 262 |
|  | 11 | 339 |
|  | 14 | 422 |
|  | 17 | 529 |
|  | 21 | 659 |
|  | 24 | 749 |
|  | 29 | 896 |
| 3 | 0 | 23 |
|  | 1 | 47 |
|  | 2 | 71 |
|  | 3 | 94 |
|  | 4 | 118 |
|  | 5 | 143 |
|  | 6 | 170 |
|  | 7 | 195 |
|  | 9 | 238 |
|  | 13 | 337 |
| 4 | 0 | 20 |
|  | 1 | 39 |
|  | 2 | 60 |
|  | 3 | 75 |
|  | 4 | 88 |
|  | 5 | 104 |
|  | 6 | 121 |
|  | 7 | 139 |
|  | 9 | 175 |
|  | 11 | 207 |
|  | 15 | 260 |
|  | 19 | 310 |
|  | 21 | 339 |
|  | 23 | 368 |
|  | 25 | 399 |
|  | 27 | 430 |
|  | 35 | 545 |
|  | 53 | 761 |
|  | 58 | 815 |
|  | 63 | 877 |
|  | 68 | 931 |
|  | 95 | 1233 |
|  | 100 | 1293 |
|  | 110 | 1409 |
|  | 120 | 1533 |
| 5 | 0 | 14 |
|  | 1 | 28 |
|  | 2 | 44 |
|  | 3 | 60 |
|  | 4 | 75 |
|  | 5 | 91 |
|  | 6 | 107 |
|  | 7 | 124 |
|  | 9 | 154 |
|  | 13 | 206 |
|  | 15 | 226 |
|  | 20 | 288 |
|  | 23 | 320 |
|  | 25 | 346 |
|  | 27 | 372 |
|  | 29 | 400 |
|  | 31 | 422 |
|  | 36 | 476 |
|  | 41 | 537 |
|  | 46 | 591 |
|  | 52 | 661 |
|  | 63 | 784 |
|  | 68 | 837 |
|  | 78 | 954 |
|  | 83 | 1008 |
|  | 88 | 1069 |
|  | 93 | 1125 |
|  | 98 | 1194 |
|  | 103 | 1275 |
|  | 113 | 1422 |
|  | 118 | 1492 |
| 6 | 1 | 23 |
|  | 2 | 35 |
|  | 3 | 49 |
|  | 4 | 62 |
|  | 6 | 91 |
|  | 7 | 103 |
|  | 9 | 125 |
|  | 11 | 152 |
|  | 13 | 170 |
|  | 15 | 189 |
|  | 17 | 213 |
|  | 19 | 240 |
|  | 21 | 265 |
|  | 23 | 286 |
|  | 25 | 302 |
|  | 27 | 326 |
|  | 29 | 352 |
|  | 31 | 376 |
|  | 36 | 433 |
|  | 41 | 487 |
|  | 46 | 540 |
|  | 51 | 597 |
|  | 66 | 772 |
|  | 71 | 828 |
|  | 76 | 897 |
|  | 82 | 977 |
|  | 87 | 1043 |
|  | 92 | 1125 |
|  | 97 | 1195 |
|  | 102 | 1272 |
|  | 107 | 1359 |
|  | 112 | 1441 |
|  | 117 | 1525 |
| 7 | 0 | 22 |
|  | 1 | 39 |
|  | 2 | 59 |
|  | 3 | 80 |
|  | 5 | 125 |
|  | 6 | 146 |
|  | 9 | 211 |
|  | 13 | 304 |
|  | 18 | 411 |
|  | 20 | 453 |
|  | 22 | 499 |
|  | 24 | 539 |
| 8 | 0 | 20 |
|  | 1 | 42 |
|  | 2 | 66 |
|  | 3 | 87 |
|  | 4 | 107 |
|  | 5 | 129 |
|  | 8 | 199 |
|  | 10 | 240 |
| 9 | 0 | 19 |
|  | 1 | 36 |
|  | 2 | 54 |
|  | 3 | 72 |
|  | 4 | 92 |
|  | 5 | 112 |
|  | 6 | 133 |
|  | 7 | 155 |
|  | 9 | 194 |
|  | 14 | 282 |
|  | 16 | 318 |
|  | 18 | 352 |
|  | 20 | 384 |
|  | 22 | 421 |
|  | 24 | 456 |
|  | 26 | 487 |
|  | 35 | 651 |
|  | 40 | 716 |
|  | 45 | 791 |
|  | 50 | 847 |
|  | 55 | 916 |
|  | 60 | 986 |
|  | 75 | 1167 |

Table S2: List of bacterial strains included in the database against which ion mobility data independent acquisition data was searched.

| Bacterial strain (taxonomy ID) | Number of proteins included (reviewed) |
| --- | --- |
| Clostridium (1485) | 5,940 |
| Pseudomonas (286) | 10,325 |
| Staphylococcus (1279) | 13,747 |
| Streptococcus (1301) | 12,759 |
| Haemophilus parainfluenza (729) | 10 |
| Alloiococcus otitis (1652) | 0 |
| Corynebacterium (1716) | 2,368 |
| Dialister (39948) | 0 |
| Finegoldia (150022) | 172 |
| Gemella (1378) | 0 |
| Peptoniphilus (162289) | 5 |
| Veillonella (29465) | 7 |
| Prevotella (838) | 15 |
| Fusobacterium (848) | 370 |
| Actinomyces (1654) | 8 |
| Granulicatella (117563) | 0 |
| Porphyromonas (836) | 528 |
| Lactobacillus (1578) | 1,575 |
| Bifidobacterium (1678) | 1,056 |
| Proteus (583) | 598 |
| Serratia (836) | 836 |
| Alcaligenes (507) | 38 |
| Enterobacter (547) | 773 |
| Bacillus (1386) | 13,948 |
| Nocardia (1817) | 404 |
| Cellulomonas (1701) | 15 |


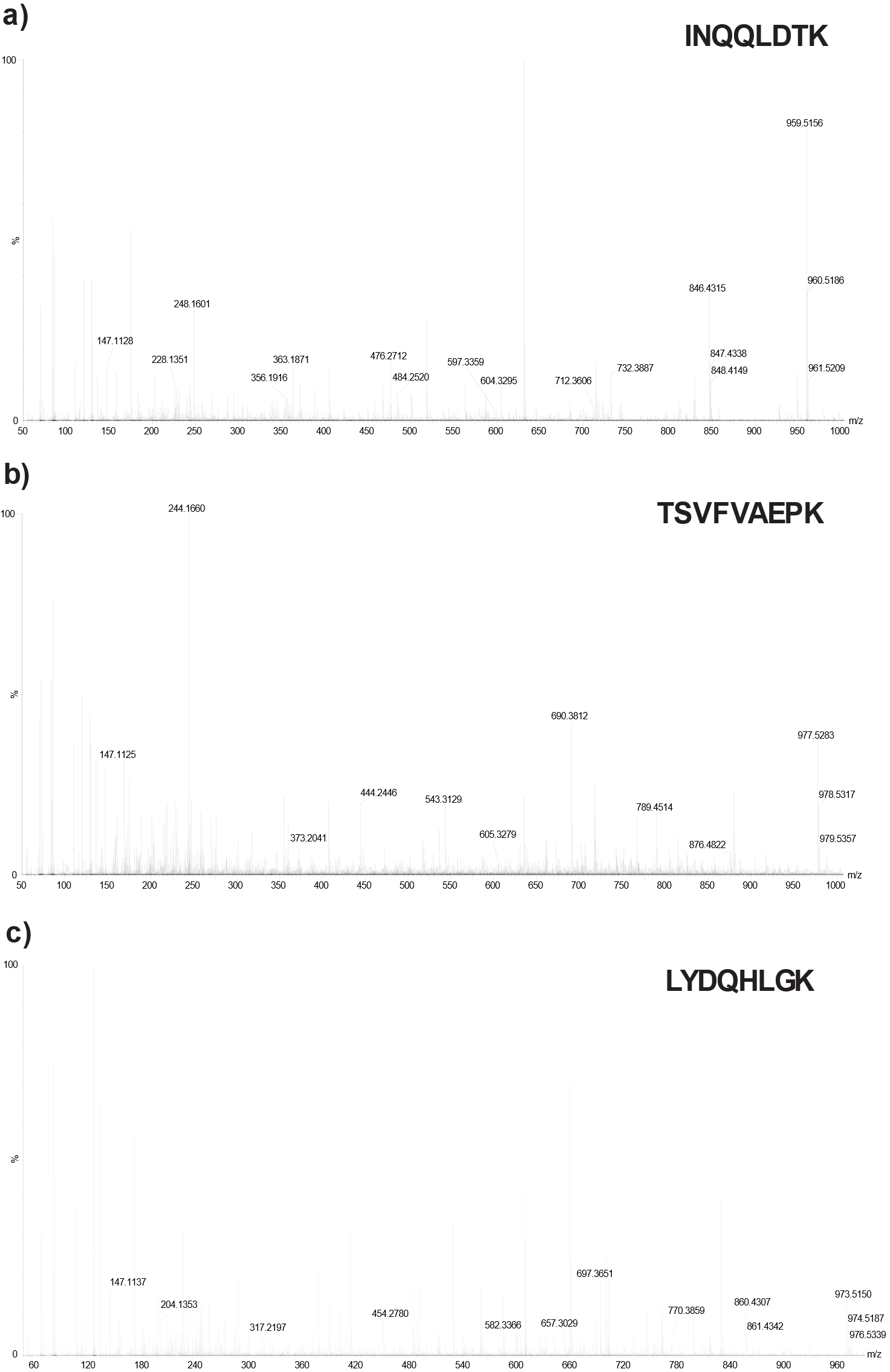
Figure S1: High energy annotated mass spectra for peptides a) INQQLDTK, RT 15.480; b) TSVFVAEPK, RT 24.448 and c) LYDQHLGK, RT 16.473; intensity normalised to largest peak.
